# Treatment with Kinase Inhibitors Plus Myo-Inositol as Re-Differentiating Agents in Iodine-Refractory Thyroid Cancers

**DOI:** 10.3390/life16030391

**Published:** 2026-02-28

**Authors:** Carlotta Giani, Michele Russo, Paola Lapi, Maria Antonietta Profilo, Raffaella Forleo, Barbara Mazzi, Arianna Ghirri, Lisa Caresia, Alfredo Campennì, Cosimo Durante, Andrea Corsello, Riccardo Morganti, Vittorio Unfer, Rosa Maria Paragliola, Daniele Barbaro

**Affiliations:** 1 Unit of Endocrinology, Azienda Usl-Toscana Nord-Ovest, 57121 Livorno, Italy; carlotta.giani@uslnordovest.toscana.it (C.G.); paola.lapi@uslnordovest.toscana.it (P.L.); mariaantonietta.profilo@uslnordovest.toscana.it (M.A.P.); raffaella.forleo@uslnordovest.toscana.it (R.F.); barbara.mazzi@uslnordovest.toscana.it (B.M.); arianna.ghirri@uslnordovest.toscana.it (A.G.); lisa.caresia@uslnordovest.toscana.it (L.C.); 2 R&D Department, Lo.Li. Pharma, Via Sabatino Gianni 14, 00156 Rome, Italy; 3 Department of Clinical and Experimental Medicine, Endocrinology Unit, University of Pisa, 56126 Pisa, Italy; 4 Department of Biomedical and Dental Sciences and Morpho-Functional Imaging, Unit of Nuclear Medicine, University of Messina, 98125 Messina, Italy; acampenni@unime.it; 5 Department of Translational and Precision Medicine, Sapienza University of Rome, 00161 Rome, Italy; cosimo.durante@uniroma1.it; 6 Unit of Endocrine Surgery, Ospedale Isola Tiberina-Gemelli Isola, 00186 Rome, Italy; andrea.corsello@fbf-isola.it; 7 Section of Statistics, University Hospital of Pisa, 56126 Pisa, Italy; r.morganti@ao-pisa.toscana.it; 8 Group on Inositol in Basic and Clinical Research and on PCOS (EGOI-PCOS), 00161 Rome, Italy; vittorio.unfer@unicamillus.org; 9 Department of Gynecology and Obstetrics, UniCamillus-Saint Camillus International University of Health Sciences, 00131 Rome, Italy; 10 Departmental Faculty of Medicine, UniCamillus-Saint Camillus International University of Health Sciences, 00131 Rome, Italy; rosamaria.paragliola@unicamillus.org

**Keywords:** myo-inositol, thyroid cancer, radioiodine refractory, kinase inhibitors, lenvatinib, sorafenib, vemurafenib, dabrafenib, selumetinib

## Abstract

Background and aim: Recent preclinical studies have confirmed that inhibiting the MAP kinase pathway can induce the re-differentiation of radioiodine (RAI)-refractory (RAIR) follicular cell thyroid cancers (TCs). The aim of this trial is to investigate whether the combination of kinase inhibitors (KIs) with myo-inositol (MI) can induce or potentiate the re-uptake of RAI in cancer cells. Overview and methods: This is an open label, non-pharmacological, multicenter, randomized pilot study. Patients will be divided into two groups: (1) a control group in which patients are treated with KIs (subgroup a: trametinib plus dabrafenib; subgroup b: lenvatinib); (2) a group in which patients (divided into the two subgroups) are treated with the same KIs in addition to MI. After 30 days of MI treatment, all patients, treated with levothyroxine (L-T4) at a semi-suppressive dosage as per clinical practice, will be stimulated with recombinant human TSH (rhTSH) (days 31 and 32). On day 35, the patients will be subjected to whole-body scintigraphy, with hybrid imaging where possible (SPECT/CT), after the administration of diagnostic activity (185–222 MBq of 123-I in accordance with the SNMMI/EANM guidelines. Blood samples will be collected before starting MI therapy (day 0); after 30 days of MI therapy; and then on days 31, 32, 33, 34, and 35 after MI therapy. Quality of life (QoL) will be assessed at the beginning of the MI treatment and at the end of its administration. The primary endpoint is the restoration of 123-I uptake in RAIR-TC patients already on KI therapy alone and on KI therapy plus MI. The restoration of 123-I uptake in target lesions will be evaluated. Conclusions: MI may have a synergistic effect at the cellular level, and the possible increase in the re-differentiation of RAIR-TC in patients treated with KIs plus MI may have great clinical relevance. The re-uptake of RAI will be evaluated as the primary endpoint, and Tg values and QoL will be evaluated as the secondary endpoints. The main limitation of this study is that we do not investigate any clinical effects. We will have to postpone the clinical analysis to a later date after the administration of RAI for therapeutic purposes.

## 1. Introduction

Thyroid cancer (TC) is the most common endocrine malignancy, and follicular cell TC is the most frequent form [1]. Human samples and animal models have led to the identification of early driver mutations (mainly BRAF V600E and RAS family), late event changes, and more recently new potential players that favor cancer transformation and/or progression [2]. At the molecular level, the Mitogen-Activated Protein Kinase (MAPK) and Phosphatidylinositol 3-kinase/Protein Kinase B (PI3K/Akt) pathways are usually dysregulated, accompanied by impaired expression of the Sodium/Iodine Symporter (NIS). Follicular cell-derived TC, with the exception of poorly differentiated TC and anaplastic TC, conserves some NIS expression in the basal plasma cell membrane. The NIS amasses iodine in the thyroid cell by transporting two sodium ions and one iodine ion into the cytosol, and iodine is then incorporated and stored in thyroglobulin, which is referred to as organification. Radioiodine (RAI) thus represents one of the first theragnostic agents in medicine. RAI is the cornerstone of treatment, after total thyroidectomy (TT), for adjuvant purposes in intermediate- and high-risk differentiated follicular cell-derived TC. RAI is also the main therapeutic option in the metastatic disease, which occurs in fewer than 10% of cases but represents the most frequent cause of TC-related death. However, unfortunately, a percentage of these metastatic cancers can become RAI-refractory (RAIR), and although rare, metastasis can be RAIR even from the initial diagnosis [3,4,5,6]. Although there is no universal definition of RAIR, RAIR-TCs have been classified into four categories, as shown in Supplementary Table S1.

The possible choice of therapeutic strategies for RAIR-TC is completely different. Local therapy, primarily surgery, is implemented where possible, but in the case of significant and diffuse progression of widespread metastatic disease, only systemic therapy offers a reasonable chance of success. An effective systemic therapy for RAIR-TC was a long-unmet need, but in the last few years, protein kinase inhibitors (KIs) have represented a turning point for the treatment of these tumors [7,8] by improving both progression-free and overall survival. In addition, as discussed below, treatment with KIs has been shown to restore some differentiation in RAIR-TC.

Protein kinases catalyze the transfer of the phosphate group from a nucleoside triphosphate donor to target proteins, resulting in a conformational change in the protein, which alters its function. Protein kinases are frequently involved in signal transduction (membrane signals, i.e., receptors) or in post-receptor cascade activation with the regulation of growth, cell cycle, apoptosis, and differentiation. Notably, among all these complex intracellular signal transduction pathways mediated by protein kinases, the MAPK and PI3K/Akt pathways are well known to be implicated in thyroid tumorigenesis. KIs are small molecules that, through different mechanisms, can interfere with the active phosphorylating domain of protein kinases, thus blocking their enzymatic function and the entire post-receptor cascade.

Several studies have demonstrated that MAPK pathway activation is associated with dedifferentiation and, in particular, NIS repression. The Cancer Genome Atlas (TCGA) Research Network highlighted that BRAFV600E-mutated papillary TC (PTC) (which has the strongest activation of the MAPK pathway) shows the most dedifferentiated state, i.e., a low expression of some thyroid differentiation genes such as the gene encoding the NIS (SCLC5A5), thyroglobulin (Tg), or thyroid peroxidase (TPO). The anti-proliferative effects of KIs may thus re-induce the ability to take up iodine, and the possible role of re-differentiation is currently under investigation in several trials.

Several in vitro studies have demonstrated an increase in NIS expression and/or iodine uptake in human TC-derived cell lines with different MAPK pathway KIs. These were, specifically, selective KI inhibitors (sKIs), namely BRAFV600E sKIs, such as vemurafenib [9,10] and dabrafenib [11]; MEK inhibitors, such as selumetinib [11,12]; and multi KIs (mKIs), such as cabozantinib [13] and sorafenib [12,13].

The first clinical phase II study to test the mKI sorafenib reported that, of the 20 patients evaluated for re-differentiation, only one showed a weak restored uptake of RAI in an occipital skeletal metastasis on a diagnostic whole-body scan (WBS); however, this was not confirmed on the therapeutic WBS [14]. Renewed interest in the re-differentiation strategy arose after a study by the James Fagin laboratory (Memorial Sloan-Kettering Cancer Center) [15]. In this latter study, an ingenious mouse model expressed the BRAFV600E oncogene in thyrocytes, and the expression of the BRAFV600E could be switched on or off with the administration or withdrawal of doxycycline. A MEK inhibitor (selumetinib) or BRAFV600E inhibitor (dabrafenib) partially restored thyroid-specific gene expression and RAI uptake. Following this preclinical in vivo study, a pioneer study by the Memorial Sloan-Kettering Cancer Center by Ho et al. in 24 patients with PTC or follicular-TC (FTC) or PDTC confirmed the ability of selumetinib to restore RAI uptake in 44% of the BRAFV600E-mutated DTC patients, who were then treated with 131I, resulting in partial tumor responses in 11% of these patients [16].

More recently, a considerable number of preclinical studies have confirmed that inhibiting the MAP kinase pathway can induce re-differentiation and tumor responses in BRAFV600E-mutated TC with clinical benefits [17,18,19,20,21]. Overall, these studies show that cellular de-differentiation may be reversible in terms of both the re-uptake of iodine and the synthesis of specific proteins such as thyroglobulin. However, during treatment, on-target and off-target mutations may be a potential general mechanism of resistance. Most in vitro and in vivo studies have used sKIs; however, some studies have also shown that cabozantinib and lenvatinib increased RAI uptake [13,22,23]. This is not surprising considering that, although mKIs have multiple targets, and although the main effect of lenvatinib is on vascular endothelial growth factor receptor (VEGFR) 1–3, this drug also interacts with platelet-derived growth factor receptor (PDGFR) and the PI3K/Akt pathway.

The literature data also suggest that myo-inositol (MI) may play a role in potentiating the action of KIs at various levels.

MI is a carbocyclic polyol and belongs to the inositol (IS) family, which consists of nine possible structural isomers. MI is the IS most widely distributed in nature and is present in fresh fruits, vegetables, cereals, legumes, and nuts. However, MI is also endogenously synthesized from glucose-6-phosphate and, in some tissues, represents approximately 99% of intracellular IS. MI is a fundamental component of structural lipids in cell membranes such as phosphatidylinositol (PI) and different phosphatidylinositol phosphates (PIPs), which are precursors for many other IS-containing compounds involved in signal transduction, vesicle trafficking, cell differentiation, and growth.

The generation of MI and its intracellular derivates is partly under the control of TSH. In fact, at slightly higher physiological concentrations, TSH activates the phospholipase C-dependent inositol phosphate Ca^2+^/diacylglycerol (DAG) pathway, with the formation of mainly inositol 1,4,5-triphosphate (IP3). IP3 increases the concentration of intracellular Ca^2+^ by favoring its release from the endoplasmic reticulum. Calcium is essential for DUOX2 activation and H_2_O_2_ generation and activates the entire DUOX2/TPO complex, iodine organification, and thyroid hormone synthesis [24,25]. In addition, other IS polyphosphates (3.4 and 3.4.5 polyphosphates) can inactivate Akt, which is a serine/threonine-specific protein kinase also known as protein kinase B. Because activating mutations in proteins of the PI3K/Akt/mTOR pathways is implicated in tumorigenesis and the dedifferentiation of thyroid cells, the abovementioned IS polyphosphates may play a protective (anti-tumoral) role and specifically increase tumor re-differentiation [26]. The last intriguing potential effect of the IS family in potentiating iodine uptake in thyroid cells is the positive role of PIP5K in the recruitment and activation of EZRIN, which binds to NIS and increases its membrane residency [27].

In summary, MI may increase the uptake and retention of iodine inside the tumor cell and block the dedifferentiation pathway, thus acting in a synergistic fashion with KIs. The aim of this trial is thus to investigate whether the combination of MI with sKIs and mKIs can induce or potentiate the re-uptake of RAI in cancer cells. Moreover, our protocol regarding the use of KIs as re-differentiating agents differs significantly from most other protocols.

## 2. Materials and Methods

### 2.1. Overview of Trial Design

This is an open-label, non-pharmacological, multicenter, randomized pilot study. Data will be collected on RAIR-TC patients being treated with KIs as per clinical practice in multiple institutions across Italy. The protocol was approved by the Ethics Committee (UniCamillus, Rome E00114-2025). The enrollment of patients will start after the protocol’s approval by the ethics committee of each center and will continue until the target sample size has been achieved (see below). The site personnel will capture all the data (epidemiological, clinical, pathological, and biochemical) electronically at the study site in an Excel database provided by the coordinating center.

The MI supplement will be shipped to the hospital pharmacy of the coordinating center and satellite centers. The product will be delivered by the pharmacy to the investigator at each center, who will then dispense it to the patients enrolled in the MI treatment group. The product can be stored at room temperature.

### 2.2. Eligibility Criteria and Study Design

Patients meeting the eligibility criteria described in Supplementary Table S2 will be included. In summary, RAIR-TC patients will need to have had next-generation sequencing (NGS) analysis for possible mutations. Eligible patients will have a BRAF mutation (treated with sKIs), no mutations, or non-targetable mutations (treated with mKIs). Patients must have been taking KIs for at least four months.

All patients will need to give their written informed consent to be included in the study.

**Figure 1:**
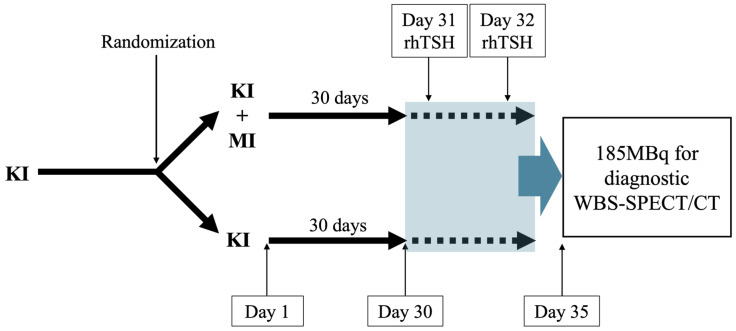
Study design. KI, Kinase Inhibitor; MI, Myo-Inositol; rhTSH, Recombinant Human TSH; MBq, Mega-becquerel (accessed on 13 January 2026); WBS-SPECT/CT, Whole-body scan-Single Photon Emission Computed Tomography/Computed Tomography.

The trial’s design is summarized in Figure 1. Patients will be divided into 2 groups: (1) a control group in which patients are treated with KIs (subgroup a: trametinib plus dabrafenib; subgroup b: lenvatinib); (2) a group (divided into the same subgroups), in which patients are treated with the same KIs in addition to MI. Comparisons will be made between the same subgroups.

MI has a half-life of less than 24 h, and a complete steady state can be achieved in about 4–5 days. However, a longer administration (30 days) helps to ensure an effect on the various metabolic activities. After 30 days of MI treatment, all patients, treated with L-T4 at a semi-suppressive dosage as per clinical practice, will be stimulated with recombinant human TSH (rhTSH) (days 31 and 32). On day 35, patients will be subjected to WBS, with hybrid imaging where possible (SPECT/TC), after the administration of diagnostic activity (185–222 MBq) of 123-I in accordance with the SNMMI/EANM guidelines [28]. Blood samples will be collected before starting MI therapy (day 0); after 30 days of MI therapy; and then on days 31, 32, 33, 34, and 35 after MI therapy. The laboratory assessments are reported in Supplementary Table S3. Quality of life (QoL) will be assessed at the beginning of the MI treatment and at the end of its administration.

Patients will be withdrawn from the study if they experience any side effects from the MI or if they experience adverse unmanageable effects from the KIs, although no known side effects of MI have been reported to date.

The study will be terminated early if there is premature evidence of superiority in the MI group. The study will conclude upon evaluation of the primary endpoint.

### 2.3. Sample Size and Procedure

We estimate that the difference between the two percentages of subjects in terms of iodine uptake at the end of treatment will equal 30% (for example, 60% in the experimental group and 30% in the control group). We also set the alpha error and power at 5% and 80%, respectively. Given these conditions, 42 patients per group will be necessary for the effect size indicated above to lead to a statistically significant result. A total of 84 patients will therefore need to be enrolled. A chi-square test will be used for the primary endpoint. Univariate and multivariate tests will be used for secondary endpoints. A multivariate logistic regression will be performed adjusting for gender and age.

The patients will be enrolled with a 1:1 ratio in either the control (KI group) or treatment MI (KI plus MI group) groups. The randomization list will be generated using Excel. We will include RAIR-TC patients being treated with KIs as per clinical practice. The enrolled patients will be divided into groups based on the KI type and then into two subgroups: KIs only vs. KIs plus MI. All patients will need to give their written informed consent to be included in the study. If not already available for all patients, an NGS molecular panel will be performed to look for somatic mutations. Each patient will be identified by a two-digit code and patient initials, which are the only identification elements and will only be used for the purposes of the study. Clinical data will be collected during the randomization, on the first (Day 1) and last day (Day 30) of the supplementation with MI (in both the KI and KI plus MI groups), and then on the day of the whole-body scintigraphy (Day 35).

### 2.4. Study Aims

The objective of the study is to evaluate the efficacy of 2 g of MI twice a day for RAIR-TC in inducing the re-uptake of RAI in patients during KI therapy. Although evaluating the effectiveness of the chronic treatment of KIs on re-differentiation is not one of the endpoints of the study, some possible speculations are discussed below.

The primary endpoint is the restoration of 123-I uptake in RAIR-TC patients already on KI therapy alone and on KI therapy plus MI. The restoration of 123-I uptake will be evaluated in target lesions. In the case of multiple lesions, only re-uptake in a target lesion will be considered. A regional target/background ratio of more than 4 and a 2-fold higher iodine uptake than the mean uptake in liver parenchyma according to visual assessment will be considered to indicate responders.

The secondary endpoints are (1) the thyroglobulin trend as a secondary marker of differentiation and (2) the safety and QoL of these patients.

Safety will be assessed according to the clinical symptoms and laboratory parameters. This includes the monitoring of adverse events as defined in the Common Terminology Criteria for Adverse Events (CTCAE) v5.0. All the investigators involved in the study will be responsible for reporting all adverse events occurring during the study.

### 2.5. Data Collection

Demographic data, such as age, gender, race, ethnicity, medical history, TNM stage at initial diagnosis, metastatic disease status, type of metastatic site, and number of organ metastases, will be collected and recorded. Data on TNM, pathological subtype, and somatic molecular mutations will also be collected. History of previous treatments for TC, such as type of surgical treatment, radiotherapy, other KI therapies, or other specific treatments (embolization, chemoembolization, thermal ablation), will be collected. Laboratory data will also be collected on a prespecified day of the study (see Supplementary Table S3).

Data on adverse events will also be collected and recorded according to CTCAE v5.0. The minimum information required is as follows:

- Data onset;
- The degree of the event’s severity;
- Whether the adverse event is serious;
- Causality with the drugs;
- Any other medical interventions performed by the investigator.

The site personnel will capture all data electronically at the study site in an Excel database provided by the coordinating center.

## 3. Discussion

In this trial, we will evaluate the potential role of the addition of MI to treatment with KIs (sKIs and mKIs) in restoring RAI uptake. From a practical point of view, we decided to investigate the most commonly used drugs: dabrafenib plus trametinib for BRAFV600E mutated cancer and lenvatinib for BRAF wild-type or other non-targetable mutated cancers.

As is well known, RAI uptake is only the first step in the therapeutic effect of RAI. The balance among RAI uptake, iodine organification, and iodine leak or its secretion in thyroid hormones determines the residence time inside the thyroid cells and, hence, the final action of RAI in terms of Gy delivered to the tissue. However, RAI uptake represents the first essential step for RAI treatment.

In our protocol, to simplify the study, we decided to only investigate RAI uptake. From an ethical point of view, we may of course administer RAI therapy in patients in which we find significant RAI uptake in lesions that previously showed no uptake, and subsequently, we will be able to investigate the effects of this treatment. In fact, we decided to evaluate the clinical effects in a second step by imaging after RAI treatment. On the other hand, the Tg values and QoL, as secondary endpoints, are measurable markers of the effects of the treatment.

In the past, various substances and drugs [29,30,31,32] that appeared to increase RAI uptake did not produce clinical effects; however, the robust literature shows that the increase in RAI uptake induced by KIs can have benefits for tumor mass in a percentage of cases.

The novel features of our trial are thus as follows:

Firstly, to the best of our knowledge, this is the first study to explore the possible role of MI in potentiating the re-differentiation of KIs in TC and the potential synergistic effects of MI. Moreover, some studies have shown a potential benefit of MI in oncology, with broad anticancer activity [33,34]. In addition, MI is a nutraceutical agent and virtually free of side effects; this is of special importance for oncological patients. Some studies also suggest improvements in the QoL of patients treated with MI. The double arm in this study will enable us to understand the respective roles of KIs alone and KIs plus MI.

In the protocol, we need to tackle the issues regarding the dose of MI and the time of administration. MI has a half-life of about 24 h and may thus reach a steady state after 4–5 days. However, we preferred a more prolonged administration period to enable the optimal incorporation of MI in the plasma membrane. Another important aspect concerns the dosage. According to the literature, a 4 g dose of MI shows significant clinical efficacy across different pathological conditions [35,36]. This is also the maximum dose allowed by the Italian Ministry of Health for use as a nutraceutical (https://www.salute.gov.it/new/sites/default/files/imported/C_17_pagineAree_1268_4_file.pdf Accessed on 21 December 2025). It is worth noting, however, that several studies have also demonstrated the effectiveness of MI at lower doses, particularly in the management of thyroid disorders [37,38,39].

In the present study, we will include α-lactalbumin in the supplement alongside MI, because this prebiotic molecule plays an important role in enhancing the intestinal absorption of MI through the gap junctions of enterocytes [40].

In addition to adding MI to the KI treatment, which is the main aim of our investigation, our protocol has several differences compared to previous protocols for the re-differentiation of TC by KIs.

In most protocols, KIs were administered for re-differentiation purposes only when the tumor was in progression, and RAI uptake was investigated shortly afterwards. For this reason, in some cases, the final clinical effects (KI itself vs. RAI therapy) on tumor mass and progression were not clearly separable.

In our protocol, patients have already been on treatment with KIs for a reasonably long time, and thus, any subsequent clinical effect of RAI therapy in patients with restored RAI uptake would be without doubt due to the effect of the latter. We decided to investigate not only patients on KI treatment with stable disease but also patients on KI treatment who experience progressive disease. We realize that these patients theoretically have a low chance of having restored RAI uptake. However, no study has investigated whether inhibition growth and RAI uptake could be separate effects of KIs.

The investigation of the effect of the therapy with lenvatinib is another novelty because, to date, few studies have investigated mKIs as re-differentiating agents.

As secondary endpoints, we will consider unstimulated Tg values. Some of the literature data show that Tg can increase together with re-differentiation [16]. It would thus be interesting to evaluate the behavior of Tg as a surrogate of re-differentiation with KIs alone and KIs plus MI.

The other secondary endpoint is QoL. As already stated, as a general presupposition, MI should not negatively impact the QoL of patients. There are some data on the use of MI regarding hypothyroidism, and the complex effects of MI on metabolism could be of help in these fragile patients. In fact, we would expect some beneficial effects on the patients’ well-being.

In the future, it would be interesting to also evaluate whether several months of treatment with KIs could improve the possibility of inducing re-differentiation. Our aim was not to have a further control group in which KI was added when the disease progresses and just before RAI treatment, as in the previous studies. However indirect comparisons could be made with previous studies.

## 4. Conclusions

The study evaluates the possible re-differentiation of RAIR-TC in patients treated with KIs plus MI. The re-uptake of iodine will be evaluated as the primary endpoint, and Tg values and QoL will be evaluated as the secondary endpoints. The main limitation of this study is that we do not investigate any clinical effects. However, we will have to postpone the clinical analysis to a later date after the administration of RAI for therapeutic purposes. The choice of 123-I was just to prevent the stunning effect. We believe that this study presents novel aspects that could lead to new forms of treatment.

## Data Availability

The raw data supporting the conclusions of this article will be made available by the authors on request.

## Supplementary Materials

The following supporting information can be downloaded at: https://www.mdpi.com/article/10.3390/life16030391/s1, Table S1: Criteria to define differentiated radioiodine-refractory thyroid cancer; Table S2: inclusion and exclusion criteria; Table S3: laboratory and radioiodine whole body scan schedule.

## Abbreviations

CTCAE	Common Terminology Criteria for Adverse Events
DAG	diacylglycerol
EANM	European Association of Nuclear Medicine
ECOG	Eastern Cooperative Oncology Group
FTC	Follicular thyroid cancer
IP3	1,4,5-triphosphate
IS	inositol
KIs	kinase inhibitors
L-T4	levothyroxine
MAPK	Mitogen-Activated Protein Kinase
MI	myo-inositol
mKIs	multi kinase inhibitors
NGS	next generation sequencing
NIS	Sodium/Iodide Symporter
PDGFR	platelet-derived growth factor receptor
PI	phosphatidylinositol
PI3K/Akt	Phosphatidylinositol 3-kinase/Protein Kinase B
PIPs	phosphatidylinositol phosphates
PT	prothrombin time
PTC	papillary thyroid cancer
QoL	quality of life
RAI	radioiodine
RAIR	radioiodine-refractory
RAIR-TC	radioiodine-refractory thyroid cancer
rhTSH	recombinant human TSH
sKIs	selective kinase inhibitors
SNMMI	Society of Nuclear Medicine and Molecular Imaging
SPECT/CT	Single-Photon Emission Computed Tomography-Computed Tomography
TC	thyroid cancer
TCGA	Cancer Genome Atlas
Tg	Thyroglobulin
TPO	thyroid peroxidase
TT	total thyroidectomy
VEGFR	vascular endothelial growth factor receptor
WBS	whole-body scan
