# Supplementary material for: Treatment with Kinase Inhibitors Plus Myo-Inositol as Re-Differentiating Agents in Iodine-Refractory Thyroid Cancers
Source: Life (Basel). 2026 Feb 28;16(3):391. doi: 10.3390/life16030391 (PMC13027575; doi:10.3390/life16030391)
Supplement: Supplementary file 1 [file life-16-00391-s001.zip › life-4125382-supplementary/life-4125382-supplementary.pdf]

Table S1: Criteria to define differentiated radioiodine-refractory thyroid cancer.

**1**	Malignant/metastatic structurally evident disease that is not able to concentrate RAI: no uptake outside the thyroid bed at the first post therapeutic whole-body scan.
**2**	Tumor tissue that loses the ability to concentrate RAI after previous evidence of RAI-avid disease (in the absence of stable iodine contamination), just one lesion that does not concentrate RAI can be defined as RAIR.
**3**	Metastatic disease that progresses despite the high cumulative activity of RAI.
**4**	Metastatic disease that progresses after a few months of high-dose RAI treatment.

RAI, radioiodine
RAIR, radioiodine-refractory

Table S2: Inclusion and exclusion criteria.

**Inclusion criteria**
• Signed the informed consent<br>• Patients with RAIR-TC, already undergoing KI therapy and stable disease (for at least 4 months) or in progression not susceptible to alternative therapy.<br>• ECOG performance status </= a 2<br>• Life expectancy > 6 months<br>• Any KI based on the indications provided by the molecular analysis and technical data sheets
**Exclusion criteria**
• Patients with lesions in which any volumetric increase after TSH stimulation could be dangerous<br>• ECOG performance status > 2<br>• Pregnancy<br>• Life expectancy < 6 months

RAIR-TC: Radioiodine-Refractory Thyroid Cancer
ECOG, Eastern Cooperative Oncology Group
KI, kinase inhibitors

Table S3: Laboratory and radioiodine whole body scan schedule.

**Laboratory data**	Days							
	0	15	30	31	32	33	34	35
TSH, FT4	X			X	X	X	X	X
Tg, TgAbs	X			X	X	X	X	X
Blood count	X		X					
PT, INR	X		X					
GOT, GPT, GGT	X		X					
Urinary albumin	X		X					
Overview imaging								
123-I WBS scanning + SPECT								X

Tg, thyroglobulin
TgAbs, thyroglobulin antibodies
WBS, whole-body scan
SPECT, Single-Photon Emission Computed Tomography
PT, Prothrombin Time
